# Gastric cancer fibroblasts affect the effect of immunotherapy and patient prognosis by inducing micro-vascular production

**DOI:** 10.3389/fimmu.2024.1375013

**Published:** 2024-07-08

**Authors:** Yan Xia, Xiaolu Wang, Jie Lin, Yuan Li, Lidan Dong, Xue Liang, Huai-Yu Wang, Xia Ding, Qi Wang

**Affiliations:** ^1^ National Institute of Traditional Chinese Medicine (TCM) Constitution and Preventive Medicine, Beijing University of Chinese Medicine, Beijing, China; ^2^ School of Traditional Chinese Medicine, Beijing University of Chinese Medicine, Beijing, China

**Keywords:** gastric cancer, bioinformatics, cancer-associated fibroblasts, angiogenesis, micro-vascular

## Abstract

**Introduction:**

Immunotherapy is critical for treating many cancers, and its therapeutic success is linked to the tumor microenvironment. Although anti-angiogenic drugs are used to treat gastric cancer (GC), their efficacy remains limited. Cancer-associated fibroblast (CAF)-targeted therapies complement immunotherapy; however, the lack of CAF-specific markers poses a challenge. Therefore, we developed a CAF angiogenesis prognostic score (CAPS) system to evaluate prognosis and immunotherapy response in patients with GC, aiming to improve patient stratification and treatment efficacy.

**Methods:**

We assessed patient-derived GC CAFs for promoting angiogenesis using EdU, cell cycle, apoptosis, wound healing, and angiogenesis analysis.

**Results:**

We then identified CAF-angiogenesis-associated differentially-expressed genes, leading to the development of CAPS, which included THBS1, SPARC, EDNRA, and VCAN. We used RT-qPCR to conduct gene-level validation, and eight GEO datasets and the HPA database to validate the CAPS system at the gene and protein levels. Six independent GEO datasets were utilized for validation. Overall survival time was shorter in the high- than the low-CAPS group. Immune microenvironment and immunotherapy response analysis showed that the high-CAPS group had a greater tendency toward immune escape and reduced immunotherapy efficacy than the low-CAPS group.

**Discussion:**

CAPS is closely associated with GC prognosis and immunotherapy outcomes. It is therefore an independent predictor of GC prognosis and immunotherapy efficacy.

## 1 Introduction

Gastric cancer (GC) is a common digestive system malignancy and the leading cause of cancer-related deaths worldwide, with few effective treatments (1). Immunotherapy involves a specific immune response to tumor cells, such as stimulation, inhibition, and killing, thus reducing tumor recurrence and metastasis. Advances in targeted therapy and immunotherapy have facilitated personalized GC treatment, significantly improving prognoses (2). Immunotherapy significantly improves the overall survival (OS) of patients with advanced GC (3); however, immunotherapy application for GC faces challenges, such as immune evasion, immune microenvironment complexity, and immunotherapy resistance (4). Several immunotherapeutic approaches have recently been developed, including vaccinations, monoclonal antibodies, and immune checkpoint inhibitors (ICIs) (5, 6). As immune checkpoints are associated with suppressive pathways, they are critical for tumor immune escape (7). ICI therapy has therefore emerged as a new cancer treatment (8–10). However, for most cancers, only one-third of patients respond to ICIs (1). Therefore, research on reliable biomarkers to accurately predict GC prognosis and immunotherapy efficacy is needed.

Angiogenesis is key for tumor progression, growth, and metastasis (11). Pathological angiogenesis can expand cancerous tissues, as well as promoting GC cell invasion and metastasis (12). Identifying targeted proangiogenic factors has become a research hotspot for treating tumors and preventing tumor progression (13). Vascular endothelial growth factor (VEGF) is an important target molecule for antitumor angiogenesis that has widely shown good therapeutic efficacy (14). Anti-VEGF therapy for GC has produced good clinical results, however, some patients develop refractory disease and resistance (15). Therefore, exploring other effective targets for inhibiting angiogenesis is necessary (15).

Angiogenesis in the tumor microenvironment (TME) is caused by interactions between multiple cells and factors. The TME contains not only cancer cells, but also stromal cells, new blood vessel immune cells, and the extracellular matrix (ECM), which affects tumor initiation, progression, metastasis, recurrence, and drug resistance (16).

As the most prominent cell type in the tumor stroma, cancer-associated fibroblasts (CAFs) are an important source of growth factors and cytokines that promote tumor progression and migration (17–19). Cytokines and chemokines produced by CAFs can remodel the ECM, positively regulating the immune response and angiogenesis in tumors. In turn, these contribute to immune suppression in the TME and tumor escape, leading to tumor progression and poor prognosis (20, 21).

Studies show that CAFs are essential for breast cancer progression and metastasis as they promote angiogenesis and lymphangiogenesis. By secreting SDF-1, CAFs attract endothelial progenitor cells to tumor tissues and induce tumor cells to generate VEGF to indirectly promote angiogenesis and provide nutrients for tumor growth (22, 23). CAFs can promote tumor cell metastasis to lymph nodes and promote new lymphatic vessel generation from existing lymphatic vessels, contributing to breast cancer progression. In contrast, lymphatic endothelial cells express VEGF receptor 3 (VEGFR-3), a major lymphangiogenesis regulator (24–26). However, few studies have reported whether CAFs are involved in inducing angiogenesis in GC. Therefore, researching the mechanism by which CAFs promote GC development and progression by regulating angiogenesis is important.

This study aimed to test whether human GC-CAFs can promote human umbilical vein endothelial cell (HUVEC) angiogenesis, compare the differences in clinical characteristics and prognosis between patients with high- and low-CAF levels, and construct a predictive model using CAF-angiogenesis-related genes.

## 2 Methods

**Figure 1:**
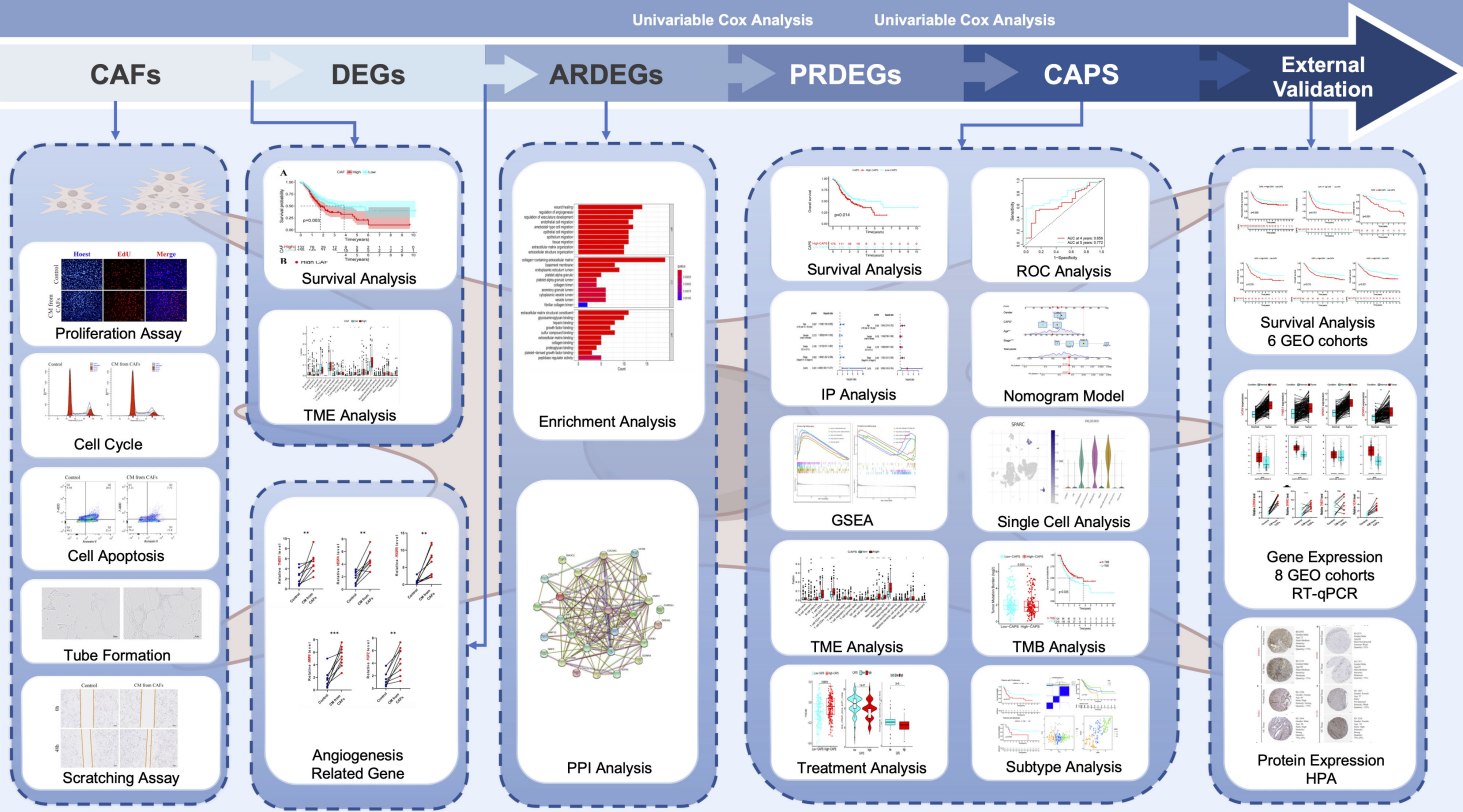
Flowchart of the data analysis process.

**Figure 1** shows the flow of this study.

### 2.1 Endothelial cell culture

HUVECs were purchased from San Diego (HTX1922, USA) and cultured in ECM medium containing 10% fetal bovine serum (FBS) for 24 h at 37°C in 5% CO_2_. They were then starved in serum-free medium for 24 h. Primary fibroblasts were extracted from GC tissue and cultured in FM2 medium containing 10% FBS. Supernatant from these fibroblasts was added to the HUVEC culture and co-cultured for 24 h to create the positive group. GES-1 cell supernatant was used in the negative control group.

### 2.2 Tube formation experiment

Matrigel, which was kept on ice, was shaken and mixed by vortex to avoid delamination. Next, 100 μL Matrigel per well was added to a precooled 24-well plate with a precooled pipette tip Matrigel to avoid bubbles Before incubation at 37°C for 45 min until the Matrigel solidified. Pretreated HUVECs were digested with trypsin, centrifuged, resuspended, and counted, and the cell suspension was adjusted to 4 × 10^5^ pieces/ml. In total, 100 μL cell suspension was added to three wells per group. After incubation at 37°C for 4 h, tubule formation was observed and photographed via optical microscope.

### 2.3 EdU assay

Cells in the logarithmic growth phase were harvested, seeded in 96-well plates at 2 × 10^4^ cells per well, and cultured until the typical growth stage. HUVECs were co-cultured for 24 h with media containing supernatants derived from GC fibroblasts or GES-1 cells. Using the EdU Cell Proliferation Detection Kit (C0071S, Beyotime, China), the cells were labeled with EdU, fixed, stained with Apollo, and counterstained with DNA dye. The cells were observed and photographed via fluorescence microscopy.

### 2.4 Cell cycle analysis

HUVECs were seeded into 6-well plates at 5 × 10^4^/mL and incubated at 37°C with 5% CO_2_ for 24 h. After incubating with supernatant from CAFs and GES-1 for 48 h, cells were harvested, fixed with 70% cold ethanol overnight at 4°C, then incubated with PI/RNaseA solution for 30 min. Flow cytometry was used for analysis.

### 2.5 Apoptosis analysis

HUVECs were seeded into 6-well plates at 5 × 10^4^/mL and incubated at 37°C and 5% CO_2_ for 24 h. Cells were treated with CAF or GES-1 supernatant for 48 h, before harvesting the cells and collecting their supernatants. AnnexinV was added and mixed well, followed by incubation in the dark at room temperature. Finally, 7-AAD and PBS were added, and the samples were analyzed immediately using flow cytometry.

### 2.6 mRNA expression level detection

HUVECs were seeded into 6-well plates at 5 × 10^4/^mL and incubated at 37°C with 5% CO_2_ for 24 h. They were then incubated with either CAF or GES-1 supernatant for 48 h before harvesting the cells. Total RNA was extracted using Trizol reagent (SM129–02, Sevenbio, China). cDNA was synthesized using a reverse transcription kit (1119ES60; Yeasen, China). RT-qPCR was performed using a SYBR Green Master Mix kit (11184ES03, Yeasen, China) and an RT-qPCR machine (SLAN-96p, Shanghai Hongshi, China). Glyceraldehyde-3-phosphate dehydrogenase served as the internal control. The 2^-ΔΔCt^ method was used for quantification. Primer sequences are provided in **Supplementary Table 1**.

### 2.7 Data collection and preprocessing

RNA-seq (FPKM format), gene mutation, and the clinical information of the TCGA-STAD cohort were downloaded from TCGA. Copy number variation (CNV) files were derived from UCSC Xena. We obtained the CAF and TIDE scores of patients with GC using the TIDE algorithm. Angiogenesis-related genes were derived from gene cards, and a relevance score > 2 was the screening criterion. The expression data and clinical files of validation cohorts (excluding patients with a follow-up time of 0) were derived from the GEO database. The clinical information of seven cohorts is shown in **Supplementary Table 2**. We also searched the GSE29272, GSE30727, GSE13911, GSE118916, GSE27342, GSE65801, GSE33335, GSE54129, and GEPIA databases using non-tumor tissues as control groups to verify model gene expression.

### 2.8 Difference analysis between high- and low-CAF groups

We used logFC > 1 and FDR< 0.05 as filters to find differentially expressed genes (DEGs) between the two groups and showed them on a volcano plot. A Venn diagram was drawn to show 33 overlapping DEGs and angiogenesis-related genes.

### 2.9 Enrichment analysis

We performed GSVA and plotted heatmaps. LogFC > 0.1 and FDR< 0.05 were considered statistically significant. We performed Gene Ontology (GO) and Kyoto Encyclopedia of Genes and Genomes (KEGG) enrichment analysis on angiogenesis-related DEGs (ARDEGs) in the high- and low-CAF groups. The filtering conditions were *P*< 0.05 and FDR< 0.05, and a histogram was drawn. Geneset files in GSVA and GSEA were obtained from the Molecular Signatures Database.

### 2.10 Protein-protein interaction (PPI) analysis

We used the STRING tool, set the minimum required interaction score to 0.4, and hid unconnected nodes to show PPI networks.

### 2.11 CAF-angiogenesis prognostic scoring model development

First, we excluded patients with 0 follow-up time in the TCGA cohort, then performed univariate Cox regression analysis and identified 26 prognosis-associated ARDEGs. For model stability, we removed genes that expressed the opposite trend to prognosis and finally identified 13 prognosis-related genes. Somatic mutations were shown using the R package “maftools” and further analyzed their CNV. Next, lasso regression analysis was used to determine model genes and corresponding coefficients and to establish a prognostic scoring model using this formula: Equation (1).


(1) $$CAPS=\sum_{i=1}^{n}\text{expr}i*\text{coefi}$$

We divided patients with GC into high- and low-CAPS groups according to the median CAPS. Kaplan-Meier survival analysis was performed. A risk curve, survival plot, and risk heatmap were drawn using the R packages “ggextra” and “pheatmap”. Time-dependent receiver operating characteristic (ROC) curves were plotted. The R package “survival” was used to verify whether CAPS could be an independent prognostic factor through univariate and multivariate Cox regression analysis.

### 2.12 Nomogram model establishment

Nomograms are widely used for cancer prognosis (27). We constructed a nomogram model to predict the OS of patients with GC at four and five years. We supplemented time-dependent ROC and calibration curves to judge the predictive ability and stability of the nomogram. This procedure was verified using the GSE15459 cohort.

### 2.13 Protein expression data validation

To further determine model gene expression at the protein level in GC tissues, we downloaded immunohistochemical images of normal and tumor tissues using the HPA database.

### 2.14 Single-cell analysis

Single-cell data from patients with GC in the GSE134520 cohort were analyzed using the Tumor Immune Single Cell Hub (TISCH) database, which was divided into nine main cell types.

### 2.15 Immune microenvironment analysis

We used the ESTIMATE algorithm to evaluate the immune, stromal, ESTIMATE, and tumor purity scores of patients with GC. We then calculated the degree of infiltration of 22 immune cells in patients with GC using CIBERPORT-ABS and the scores of 16 immune cells and 13 immune pathways by ssGSEA. The CIBERPORT-ABS algorithm results for TCGA patients were derived from the TIMER database, and we analyzed the correlation between the model genes and CAFs using four methods (EPIC, MCPCOUNT, XCELL, and TIDE) in the TIMER online database.

### 2.16 Somatic mutation and tumor mutation burden (TMB) analysis

We drew boxplots, correlation scatter plots, and Kaplan-Meier survival curves of the high- and low-TMB groups. GC prognosis was analyzed by combining TMB with CAPS.

### 2.17 Immunotherapy and drug sensitivity analysis

We drew a boxplot of the differences in TIDE scores between high- and low-CAPS groups and a scatter plot of correlations between CAPS and TIDE scores. Violin plots were drawn to show the predicted therapeutic efficacy of IPS in high- and low-CAPS groups. IPS scores of patients with GC were downloaded from the TCIA. The immunotherapy cohort (IMvigor210) was derived from previous literature (28). The melanoma cohort (GSE78220) before anti-PD-1 treatment was derived from the GEO database. CR/PR and SD/PD were used as the response and non-response groups, respectively. Additionally, we calculated the half maximal inhibitory concentration of anti-tumor drugs using the R package “pRRophetic” and plotted a boxplot. We also downloaded a three-dimensional structure map of the drug through the PubChem database.

## 3 Results

### 3.1 GC-derived CAFs promote angiogenesis

**Figure 2:**
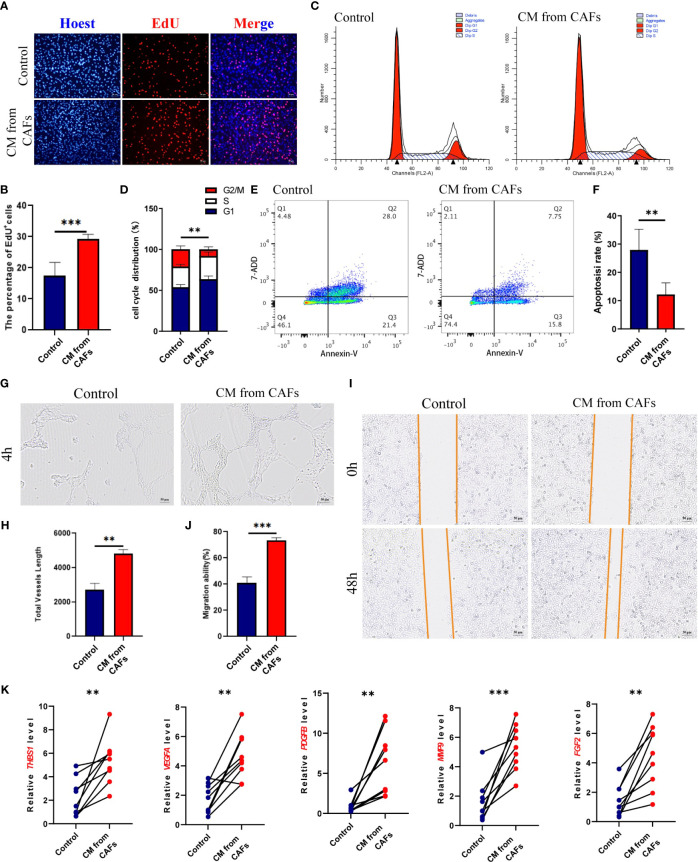
**(A, B)** HUVEC was stimulated with GES-1 and supernatant of gastric cancer (GC) fibroblasts, and the proliferation ability was detected by EDU staining. **(C, D)** Cell Cycle of HUVEC stimulated with GES-1 and supernatant from GC fibroblasts. **(E, F)** Cell Apoptosis of HUVEC stimulated with GES-1 and supernatant from GC fibroblasts. **(G, H)** Tube formation of HUVEC stimulated with GES-1 and supernatant from GC fibroblasts. **(I, J)** The migration ability of HUVEC stimulated with GES-1 and GC fibroblast supernatant was detected by wound healing assay. **(K)** The expression of *ANGPT2*, *VEGFA*, *PDGFB*, *MMP9* and *FGF2* in HUVECs stimulated with GES-1 and supernatant of GC fibroblasts was detected by RT-qPCR. **P< 0.01, ***P< 0.001.

Compared to GES-1 supernatant-induced HUVECs, CAF supernatant-induced HUVECs had a higher proliferation rate (*P*< 0.001) (**Figures 2A, B**), shorter cell cycle (*P*< 0.01) (**Figures 2C, D**), and reduced cell apoptosis (*p*< 0.01) (**Figures 2E, F**); they also had a relatively strong tube-forming ability (*P*< 0.01) (**Figures 2G, H**), high migration ability (*P*< 0.001) (**Figures 2I, J**), and upregulated expression levels of the angiogenesis-related genes *ANGPT2*, *VEGFA*, *PDGFB*, *MMP9*, and *FGF2* (**Figure 2K**). Therefore, GC-derived CAFs promote angiogenesis.

### 3.2 Different CAF levels affect patient characteristics and identifications

**Figure 3:**
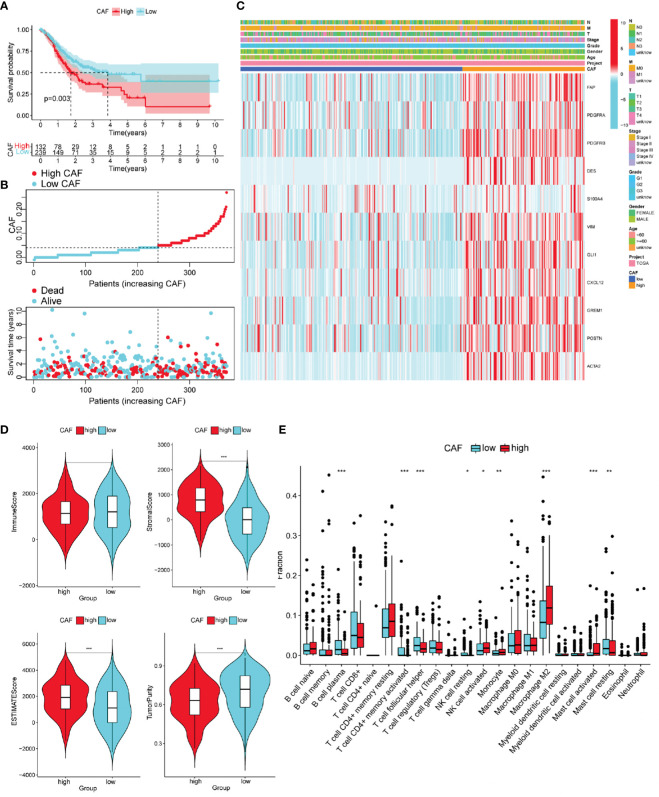
Different characteristics of GC patients with high and low Cancer-associated fibroblast (CAF) scores. **(A)** According to the optimal cut-off value (0.04), GC patients were divided into high CAF group (*n* = 132) and low CAF group (*n* = 239). Kaplan–Meier survival analysis of patients with high and low CAF scores. **(B)** Distribution of high and low CAF scores and patient survival status. **(C)** The Heatmap of CAF markers and clinical features. **(D)** The immune score, stromal score, ESTIMATE score, and tumor purity score of the two groups. **(E)** Immune cell infiltration in two groups of GC patients (CIBERPORT-ABS algorithm). *P< 0.05, **P< 0.01, ***P< 0.001.

To observe whether the degree of CAF infiltration affects the OS of patients with GC, we obtained CAF scores of patients with GC using the TIDE algorithm. OS differed significantly among the low- and high-CAF groups (**Figure 3A**). As CAF score increased, patient survival time gradually shortened (**Figure 3B**). Based on previous research (29), 11 CAF markers were selected; in the high-CAF group, these were significantly upregulated, supporting the grouping (**Figure 3C**).

We evaluated differences in GC progression between the high- and low-CAF groups in terms of the TME. The CAF group with high stromal and ESTIMATE scores was relatively higher, and the tumor purity score was lower. This is consistent with CAF being an important component of stromal cells (**Figure 3D**).

The high-CAF group had more immunosuppressive cell infiltration than the low-CAF group, including monocytes, M2 macrophages, and activated mast cells, and fewer infiltrated immune activated cells, such as plasma cells, activated CD4 memory cells, and Tfh cells (**Supplementary Figure 3E**). Another ssGSEA algorithm produced consistent results (**Supplementary Figures 1A, B**).

**Figure 4:**
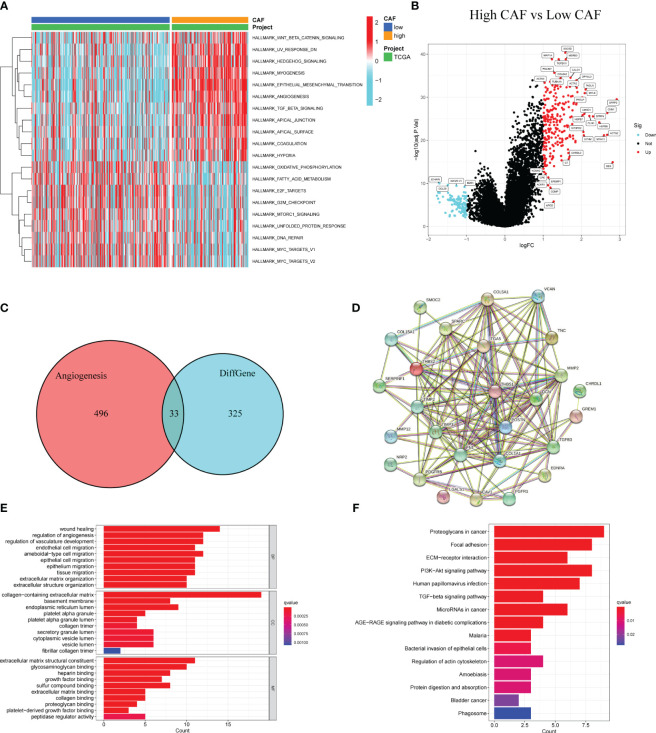
Identification of DEGs in patients with high and low CAF scores and enrichment analysis. **(A)** GSVA between high and low CAF. **(B)** The volcano plot shows DEGs (high CAF VS low CAF). **(C)** The Venn diagram shows 33 overlapping genes of DEGs and angiogenesis-related genes. **(D)** PPI analysis of 33 overlapping genes. **(E)** GO analysis of 33 overlapping genes. **(F)** KEGG analysis of 33 overlapping genes.

GSVA was used to identify differences in tumor-related characteristics between the two patient groups. Angiogenesis was upregulated in the high-CAF group (**Figure 4A**). Both groups likely regulate the immune microenvironment through angiogenesis, leading to different prognoses. We obtained 358 DEGs in the high- and low-CAF groups relating to CAF-promoting angiogenesis (**Figure 4B**). Intersection processing between the DEGs and angiogenesis-related genes produced 33 ARDEGs (**Figure 4C**). Plotting the PPIs of the 33 nodes and 142 edges showed the relationship between their protein levels (**Figure 4D**). To better understand ARDEG mechanics, we performed GO and KEGG enrichment analyses. GO analysis showed that DEGs were mainly enriched in wound healing, angiogenesis and vasculature development regulation, collagen-containing ECM, ECM structural constituents, and glycosaminoglycan and heparin binding (**Figure 4E**). KEGG analysis showed that DEGs were mainly enriched in proteoglycans in cancer, focal adhesions, and ECM-receptor interactions (**Figure 4F**). This confirms that ARDEGs are closely related to angiogenesis and vascular development.

### 3.3 CAPS as GC a prognostic indicator

**Figure 5:**
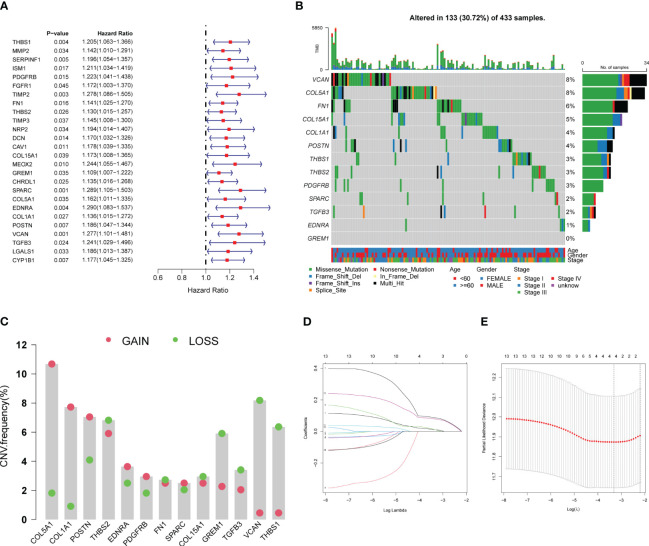
Establishment of CAF-angiogenesis prognostic scoring model. **(A)** The forest plot shows 26 prognostic-related genes. **(B)** The waterfall plot shows the mutations of 13 genes. **(C)** CNV of 13 genes. **(D)** LASSO coefficient profiles of 13 genes. **(E)** The tuning parameters were cross-validated in the LASSO model.

Univariate Cox regression analysis of the TCGA cohort revealed 26 ARDEGs, all of which were risk factors for poor GC prognosis (**Figure 5A**). To ensure model accuracy, genes with no differences in expression between GC and normal tissues or with opposite trends in prognostic risk factors were excluded. Thirteen stable candidate genes were obtained. Analysis of the somatic mutation status of GC tissues showed that 13 candidate genes were mutated in 30.72% of patients with GC, and most were missense mutations (**Figure 5B**). We also analyzed CNVs in the ARDEGs and identified the location of each gene (**Figure 5C**). Next, we established CAPS models using lasso regression analysis and performed fold-cross validation (**Figures 5D, E**). The CAPS building method was as follows: CAPS = 0.011 × thrombospondin-1 (*THBS1*) expression level + 0.086 × secreted protein acidic and rich in cysteine (*SPARC*) expression level + 0.011 × endothelin receptor type A (*EDNRA*) expression level + 0.078 × versican (*VCAN*) expression level.

**Figure 6:**
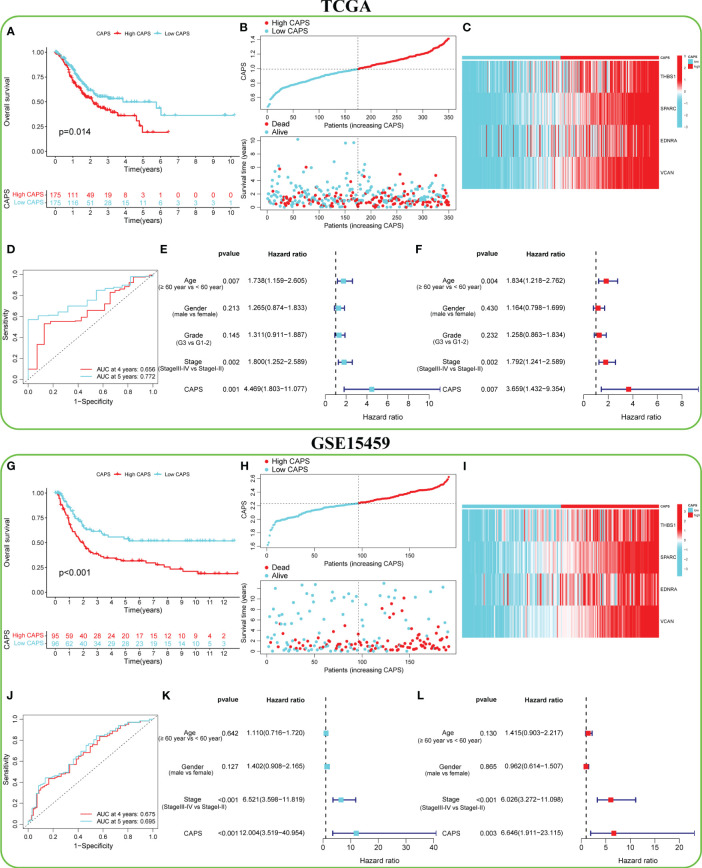
Predictive efficacy of CAF-angiogenesis prognostic score (CAPS) in the TCGA cohort and GSE15459 cohort. **(A)** Kaplan-Meier survival analysis in the TCGA cohort. **(B)** The distribution of CAPS in the TCGA cohort and the survival status of patients. **(C)** Expression levels of model genes in TCGA cohort. **(D)** The ROC curve analysis according to the 4- and 5-year survival in the TCGA cohort. **(E)** Univariable Cox analysis was used to determine the correlation with age (≥ 60 years vs< 60 years), gender (male vs female), and stage (stage III-IV vs Stage I-II) of GC patients in TCGA cohort. **(F)** Multivariable Cox analysis was used to determine the correlation with age (≥ 60 years vs< 60 years), gender (male vs female), and stage (stage III-IV vs Stage I-II) of GC patients in TCGA cohort. **(G)** Kaplan-Meier survival analysis in the GSE15459 cohort. **(H)** The distribution of CAPS in the GSE15459 cohort and the survival status of patients. **(I)** Expression levels of model genes in the GSE15459 cohort. **(J)** The ROC curve analysis according to the 4- and 5-year survival in the GSE15459 cohort. **(K)** Univariable Cox analysis was used to determine the correlation with age (≥ 60 years vs< 60 years), gender (male vs female), and stage (stage III-IV vs Stage I-II) of GC patients in the GSE15459 cohort. **(L)** Multivariable Cox analysis was used to determine the correlation with age (≥ 60 years vs< 60 years), gender (male vs female), and stage (stage III-IV vs Stage I-II) of GC patients in the GSE15459 cohort.

The TCGA cohort was divided into the high- and low-CAPS groups (*n* = 175 each) according to median CAPS. OS was lower in the high- than the low-CAPS group (**Figure 6A**). Moreover, as CAPS increased, patient survival time gradually decreased (**Figure 6B**). Model gene expression was higher in the high-CAPS group (**Figure 6C**). The AUC of the TCGA time-dependent ROC curve was 0.656 and 0.772 at 4 and 5 years, respectively (**Figure 6D**). Univariate Cox analysis showed that CAPS was a risk factor for poor prognosis of GC (**Figure 6E**). In multivariate Cox analysis, CAPS remained an independent prognostic factor for patients with GC after excluding other confounding factors (**Figure 6F**). Additionally, the four model genes were highly correlated with CAF in the four algorithms, proving that CAPS has a strong theoretical basis as a prognostic indicator (**Supplementary Figure 2**).

### 3.4 The high stability of CAPS was verified by external validation

To verify the accuracy of CAPS in predicting GC, six independent GEO datasets (GSE15459, GSE84437, GSE26901, GSE13861, GSE62254, and GSE26253) were used for external verification. In the GSE1549 cohort, for example, the OS of the high- and low-CAPS groups was significantly different (**Figure 6G**); the high-CAPS group had a shorter survival time (**Figure 6H**). Model gene expression was consistent with that in TCGA (**Figure 6I**). The AUC of the time-dependent ROC was 0.675 and 0.695 at four and five years, respectively, confirming the model’s accuracy (**Figure 6J**). CAPS was a risk factor and could be used as an independent prognostic factor (**Figures 6K, L**). The performance of CAPS in the remaining five cohorts is shown in Supplementary **Figures 3A–E**. Additionally, we found that the CAPS in the high-CAF group was significantly higher than that in the low-CAF group, with CAPS positively correlated with the CAF score (*r* = 0.66) (**Supplementary Figures 3F, G**).

### 3.5 The broad applicability of CAPS was proved by nomogram

**Figure 7:**
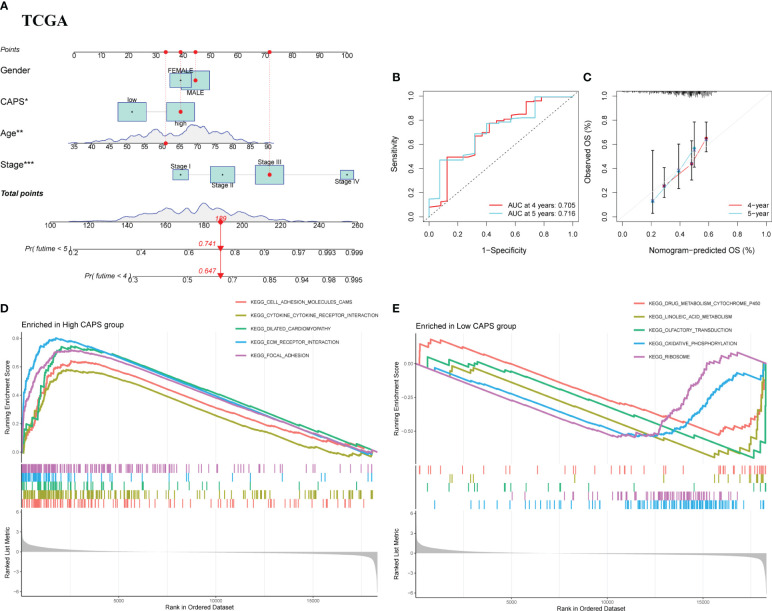
Nomograph Model and GSEA. **(A)** Nomogram of CAPS and clinical characteristics predicting survival probability of GC patients in TCGA cohort. **(B)** The ROC curve verifies the predictive ability of the nomogram in the TCGA cohort. **(C)** Calibration curve for the predictive ability of nomograms in TCGA cohort. **(D)** GSEA enrichment analysis in the high CAPS group. **(E)** GSEA enrichment analysis in the low CAPS group.

We integrated CAPS, age, sex, and pathological stage to construct and verify our nomogram. The score of each feature was calculated to obtain the total score of the predicted patient OS (**Figure 7A**). The AUC of the time-dependent ROC nomogram curve was 0.705 and 0.716 at 4 and 5 years, respectively (**Figure 7B**). We drew a calibration curve for the nomogram model, suggesting that the predicted survival time was consistent with the actual results (**Figure 7C**). Repeating this for the GSE15459 cohort demonstrated the nomogram model applicability (**Supplementary Figures 4A–C**). According to GSEA, the pathways enriched in the high-CAPS group were mainly cell adhesion molecules, cytokine receptor interactions, and focal adhesion (**Figure 7D**); those in the low-CAPS group were mainly drug metabolism, cytochrome P450, linoleic acid metabolism, and oxidative phosphorylation (**Figure 7E**).

### 3.6 CAPS was validated at both gene and protein levels

**Figure 8:**
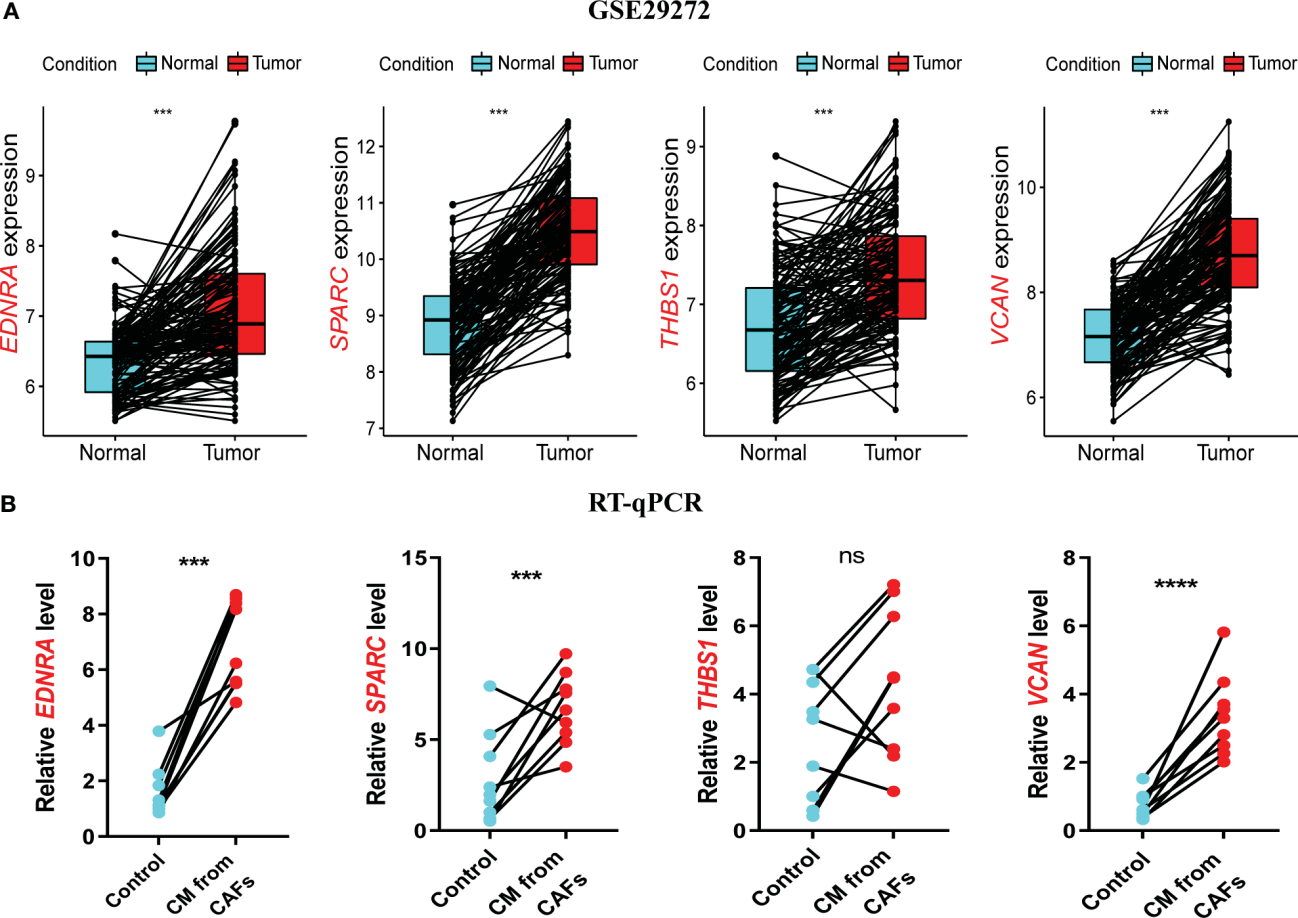
Validation of model genes at gene levels. **(A)** In the GSE29272 datasets, the expression of *Endothelin Receptor Type A* (*EDNRA)*, *Secreted protein acidic and rich in cysteine* (*SPARC)*, *Thrombospondin-1*(*THBS1)*, and *versican* (*VCAN)* in 134 adjacent normal and tumor tissues was detected. **(B)** The expression of *EDNRA*, *SPARC*, *THBS1*, and *VCAN* in HUVECs stimulated with GES-1 and supernatant of GC fibroblasts was detected by RT-qPCR. ***P< 0.001.

In the GSE29272 dataset, four model genes were significantly upregulated in the tumor group (**Figure 8A**), consistent with the other seven GEO datasets (**Supplementary Figures 5A–G**). In the GEPIA database, *EDNRA*, *SPARC*, and *VCAN* were all upregulated in the tumor group, while *THBS1* did not differ significantly, although there was an upward trend in the tumor group (**Supplementary Figure 5H**).

RT-qPCR was used to assess model gene expression levels in the CAPS system. All four genes were upregulated in CAF supernatant-treated HUVECs compared to the control group (**Figure 8B**). *THBS1* expression showed a non-significant upward trend, potentially due to the small sample size. In the HPA database, the protein expression of the four model genes EDNRA, SPARC, THBSI, and VCAN was higher in gastric cancer tissues compared with normal tissues (**Supplementary Figure 6**). In conclusion, we have demonstrated through experiments and multiple datasets that four model genes remain upregulated in GC. This upregulation has been validated at both the mRNA and protein levels, further confirming the reliable predictive ability of CAPS.

### 3.7 Single-cell analysis uncovered regulatory mechanisms of CAPS gene distribution

**Figure 9:**
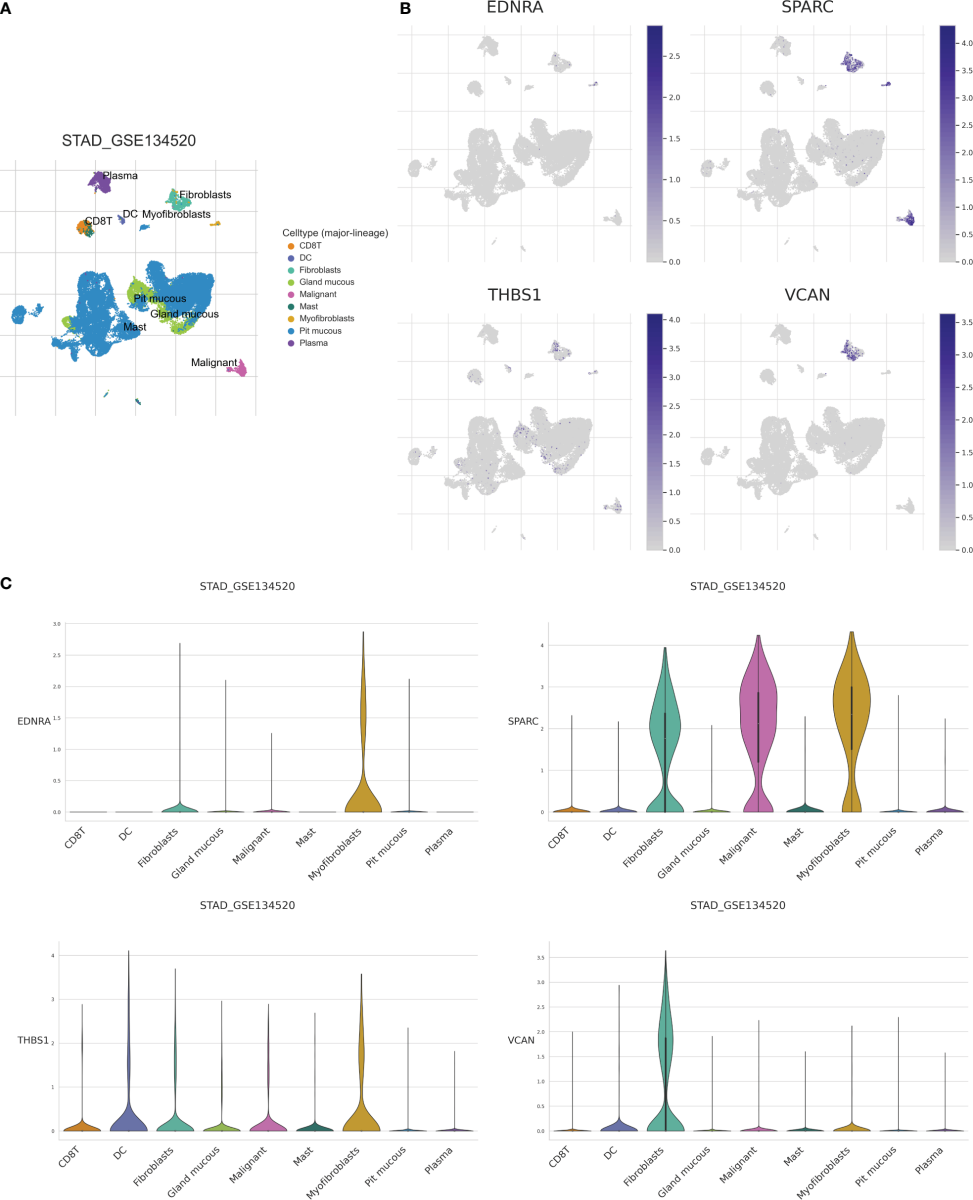
Analysis of single-cell data set (GSE134520) of GC based on the TISCH database. **(A)** CD8T, DC, Fibroblasts, Gland mucous, Malignant, Mast, Myofibroblas, Pit mucous, and Plasma as the main cell types. **(B)** Expression of model genes in each cell type. **(C)** Model genes were mainly expressed in Myofibroblas and Fibroblasts.

To further clarify model gene expression in cell subpopulations, we analyzed single-cell data from patients in the TISCH database. The nine main cell types were CD8^+^ T cells, DCs, fibroblasts, glandular mucous, malignant, mast, myofibroblastic, pit mucous, and plasma cells (**Figure 9A**). The clusters of pit mucous cells may be attributed to heterogeneity within the cell population (**Figure 9B**). *EDNRA* was mainly expressed in myofibroblasts and fibroblasts, while *SPARC* had very high expression levels in myofibroblasts, malignant cells, and fibroblasts. *THBS1* was expressed at greater levels in DCs and myofibroblasts and *VCAN* was mainly expressed in fibroblasts (**Figure 9C**). In summary, our analysis of single-cell data elucidates significant differences in the expression levels of model genes across various cell subpopulations. These findings underscore the heterogeneity within cell populations and offer valuable insights into the regulatory mechanisms of cellular function.

### 3.8 Exploring immune infiltration and responsiveness to immunotherapy between two clusters

**Figure 10:**
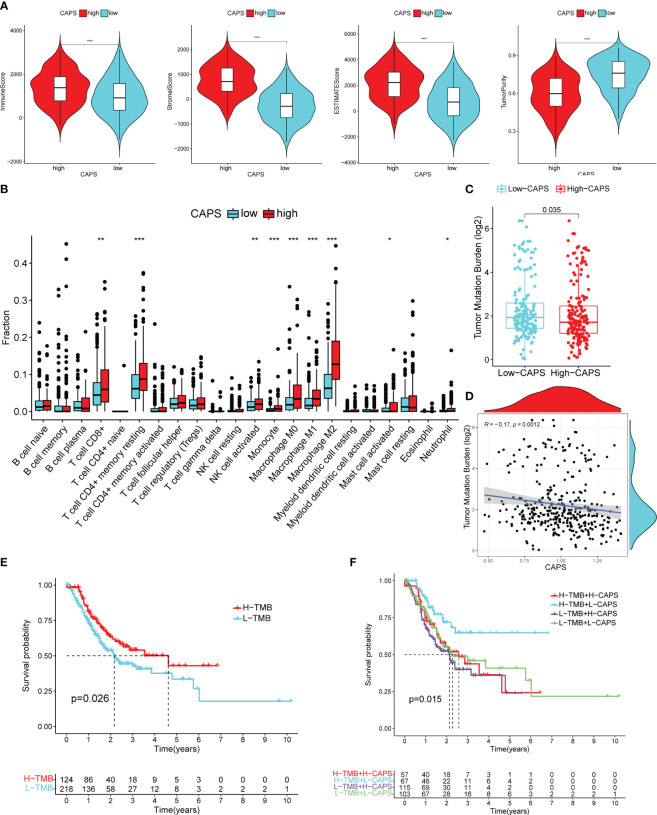
Immune microenvironment of high and low CAPS groups. **(A)** The immune score, stromal score, ESTIMATE score, and tumor purity score of the high and low CAPS groups. **(B)** Immune cell infiltration in the high and low CAPS groups of GC patients (CIBERPORT-ABS algorithm). **(C)** The boxplot of TMB in patients with high and low CAPS groups. **(D)** The scatter plot of correlation between TMB and CAPS. **(E)** Difference in survival time between high- and low-TMB groups. **(F)** Survival analysis of TMB union CAPS. *P< 0.05, **P< 0.01, ***P< 0.001.

In the high-CAPS group, the immune, stromal, and ESTIMATE scores were higher, whereas the tumor purity score was lower (**Figure 10A**). Immune cell infiltration was significantly higher in the high- than low-CAPS group, including CD8^+^ T cells, resting CD4 memory T cells, activated NK cells, monocytes, M0, M1, and M2 macrophages, activated mast cells, and neutrophils (**Figure 10B**). The high-CAPS group showed greater immune cell infiltration.

### 3.9 CAPS can predict the efficacy of immunotherapy in CG patients

TMB was higher in the low- than the high-CAPS group; TMB was negatively correlated with CAPS (*r* = -0.17) (**Figures 10C, D**). OS was better in patients with high than low TMB (**Figure 10E**). Furthermore, patients with high TMB and low CAPS had the best OS, whereas those with low TMB and high CAPS had the worst OS (**Figure 10F**). This shows that CAPS is highly consistent with TMB in assessing GC prognosis, further demonstrating the predictive performance of CAPS.

**Figure 11:**
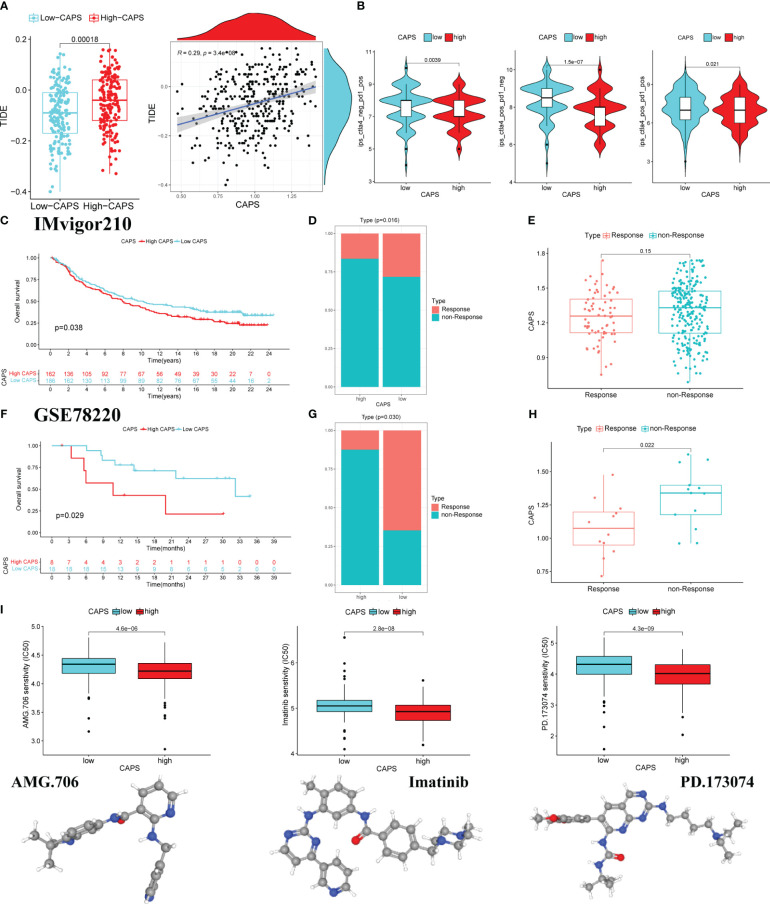
CAPS predicts the effect of immunotherapy. **(A)** The boxplot of differences in TIDE score in the low and high CAPS groups. Correlation plot of CAPS and TIDE score. **(B)** The violin plots of IPS differences between low-CAPS and high-CAPS groups. **(C)** Kaplan-Meier survival analysis in IMvigor 210 cohort. **(D)** Distribution of patients with different immunotherapy effects in the high and low CAPS groups in the IMvigor cohort. **(E)** Differences in CAPS between immunotherapy responsive and unresponsive patients in IMvigor cohort. **(F)** Kaplan-Meier survival analysis in the GSE78220 cohort. **(G)** Distribution of patients with different immunotherapy effects in the high and low CAPS groups in the GSE78220 cohort. **(H)** Differences in CAPS between immunotherapy responsive and unresponsive patients in the GSE78220 cohort. **(I)** The boxplot for predicting drug sensitivity in high and low CAPS groups.

TIDE score was used to evaluate immunotherapy efficacy. The TIDE score of the high-CAPS group was significantly higher than that of the low-CAPS group, and CAPS and TIDE scores were positively correlated (*r* = 0.29) (**Figure 11A**). This was consistent with a relatively poor immunotherapeutic effect in the high-CAPS group.

Analysis of differences in efficacy between anti-PD-1 and anti-CTLA-4, the most common ICI drugs, showed that the IPS of the low-CAPS group was higher than that of the high-CAPS group. This indicates a better immunotherapeutic effect in the low-CAPS group (**Figure 11B**).

In the IMVigor 210 cohort, OS was better in the low- than the high-CAPS group. Additionally, the proportion of patients who did not respond to treatment was higher in the high- than the low-CAPS group. Although there was no significant difference in CAPS between the treatment response and non-response groups, there was an upward trend (**Figures 11C–E**). The GSE78220 cohort produced consistent results; the low-CAPS group had better OS, there were fewer patients in the treatment-unresponsive group, and patients had a lower CAPS in the treatment-responsive group (**Figures 11F–H**). Screening for the chemotherapeutic drugs AMG.706, imatinib, and PD.173074 showed that the high-CAPS group was more sensitive (**Figure 11I**). In conclusion, CAPS provides a new impetus for the rational selection of immunotherapeutic and chemotherapeutic drugs for patients with GC.

## 4 Discussion

The TME is crucial for GC occurrence and development and tumor stromal components in the TME are essential for tumor growth and metastasis, immunosuppression, and drug resistance. CAFs are the most prominent cell type in GC stroma (30). To support the high proliferation rate of cancer cells, tumors must rapidly develop new vascular networks, leading to hypoxia, decreased immune cell infiltration and activity, and increased metastasis risk (31). CAFs can regulate angiogenesis and promote tumor progression (21), but the underlying mechanisms in patients with GC are unclear. First, we observed that CAFs promote angiogenesis in HUVECs. High-throughput data analysis showed that the high-CAF group had relatively high stromal scores, indicating a stronger immunosuppressive microenvironment and poorer GC prognosis. This demonstrates that CAFs affect the tumor immune microenvironment (TIME) by regulating angiogenesis. Therefore, we characterized the angiogenesis-related genes in CAFs to predict patient survival and immunotherapy efficacy. We developed a new CAPS system and six independent external validations were performed, as well as gene-level validation. The CAPS system accurately predicted the prognosis and immunotherapy sensitivity of patients with GC. CAPS consists of four mRNAs, all of which were consistently up-regulated in GC tissues, that is, CAPS was strongly, stably expressed at the gene and protein levels. Univariate, multivariate, and nomogram validation consistently demonstrated that CAPS independently predicted patient prognosis. Single-cell data analysis showed that the model genes were highly expressed in fibroblasts and positively correlated with CAF marker genes, further proving the authenticity, stability, and applicability of CAPS. Overall, CAPS can effectively assess the prognosis and clinical status of patients with GC. It has higher clinical applicability than previous prognostic models constructed using CAF-related genes in GC (32).

CAPS included four mRNAs, which were upregulated in GC tissues and positively correlated with poor prognosis. *THBS1* is in the thrombospondin family and is closely related to GC cell invasion and migration (33). High *THBS1* expression is an independent risk factor (34). *THBS1* can have both proangiogenic and antiangiogenic effects. *THBS1* is most commonly used as an anti-angiogenic factor, but it is positively associated with poor tumor prognosis. In ovarian cancer, IGFBP3 inhibits angiogenesis by regulating intracellular *THBS1* expression (35). *THBS1*, *THBS2*, and *PEDF* reduce angiogenesis and promote tumor-associated lymph angiogenesis in iCCA (36). However, the role of *THBS1* in GC angiogenesis is unknown. Interestingly, *THBS1* has proangiogenic activity when its N-terminal heparin-binding domain interacts with *LRP1* receptor (37, 38), showing that its role varies in different microenvironments.

*SPARC* is a secreted glycoprotein that mediates cell-ECM interactions. The function of *SPARC* relates to and varies with tumor type, cancer cell origin, and the TME. For example, high *SPARC* expression is positively correlated with poor prognosis in pancreatic cancer, invasive breast cancer, and colon adenocarcinoma (39). However, high *SPARC* expression is associated with a good prognosis in diffuse large B-cell lymphoma (40). *SPARC* is negatively associated with GC prognosis by regulating platelet activation (41). Meanwhile, *EDNRA* is an endothelin-1 receptor expressed in many malignancies that is closely associated with cell proliferation, invasion, migration, metastasis, and drug resistance (42, 43). *VCAN* belongs to the aggrecan/versican family of proteoglycans (44). Its main cellular functions are cell adhesion, proliferation, tissue morphogenesis, and maintenance (45). *VCAN* is associated with tumor growth and metastasis, including in GC (46). Overall, our model genes have a solid foundation of clinical research.

The TME is receiving increased attention due to its role in tumor immunosuppression, distant metastasis, local drug resistance, and targeted therapy responses (16). Immune cells in the TME can be used for prognostic assessment, including in GC (47). Increasing attention is also being paid to the immunosuppressive effect of CAF through interactions with TIME components, especially immune cells (48). CAFs promote cancer cell proliferation and immune escape (49). How GC-CAFs regulate the TME through angiogenesis to induce an immunosuppressive microenvironment is unknown. Therefore, we explored the correlation between CAF-angiogenesis and the GC TME.

In the high-CAPS group, immune cells were imbalanced, with higher immune cell infiltration; these cells were manipulated to protect them from the body’s immune response (50). Tumor-associated macrophages (TAMs) promote the development of an immunosuppressive TME, and can be continuously activated by the TME (51, 52). Consistent with this, TAM infiltration was relatively high in the high-CAPS group. M1 macrophages promote tumor killing, whereas M2 macrophages are associated with cancer metastasis and poor prognosis (53). However, studies show that traditional cognition is broken. CD68^+^ HLA-DR^+^ M1 macrophages rely on the NF-κB signaling pathway to promote tumor migration (54). In oral squamous cell carcinoma, M1 macrophages stimulated and polarized by exosomes promoted tumor cell migration, accelerating cancer progression (55). Different diseases and body parts affect the TME differently (56–58). In a complex TME, the molecular mechanisms associated with M1 macrophages are complex. TMB is a stable genetic marker; therefore, the expression of predicted markers is consistent with TMB and is highly reliable (59). Increased TMB is associated with a better response to immunotherapy (60). Consistent with this, TMB was higher in the low-CAPS subgroup, CAPS was negatively correlated with TMB, and patients in the high-TMB and low-CAPS groups had the longest survival time, while patients in the low-TMB and high-CAPS groups had the shortest survival time. Overall, CAPS is highly reliable for evaluating immunotherapy efficacy in GC.

TIDE is highly valuable for predicting or evaluating immunotherapy efficacy. Patients with higher TIDE scores are more likely to develop tumor immune escape, and therefore, a lower immunotherapy response rate (61, 62). Consistently, the high-CAPS group had higher TIDE scores, proving the accuracy of CAPS for evaluating immunotherapy efficacy in GC. Targeting immune checkpoints, such as PD-1 and CTLA-4, can improve antitumor immunity (63) and ICIs are effective GC treatments (64). To predict the therapeutic effect of ICIs on patients with GC, we analyzed the relationship between CAPS and PD-1 and CTLA-4 in GC. The level of anti-CTLA-4 and anti-PD-1 treatment alone or in combination was higher in the low- than the high-CAPS group, indicating that immunotherapy would be more effective in the low-CAPS group. This was confirmed in the immunotherapy cohort, with a smaller proportion of non-responsive patients in the low-CAPS group and a higher CAPS in patients in the non-responder group. Finally, we also screened therapeutic drugs to which patients in the high-CAPS group may be more sensitive. First, we confirmed the correlation between CAFs and angiogenesis. We then used CAFs and angiogenesis-related genes in TCGA sequencing data to establish a CAPS model that could predict prognosis and immunotherapy efficacy in GC, which was verified by six independent GEO datasets. Compared with the low-CAPS group, the high-CAPS group had a pro-cancer immune microenvironment, low TMB, high TIDE score, and relatively poor anti-PD-1 and anti-CTLA4 therapeutic efficacy. These findings indicate that the high-CAPS group is associated with immune escape in GC. Therefore, CAPS is a new biomarker that can effectively predict GC prognosis and immunotherapy efficacy.

This study has various limitations and further research is needed to validate the prognostic features of CAPS. The mechanism of the TME promoting CAF-vascularization characteristics in GC is unclear and will be investigated in the future. Studies on the interactions between CAFs and the immune microenvironment, especially complex mechanisms linking CAFs to immune cells, may provide new strategies for targeted immunotherapies.

Overall, we found that GC fibroblasts can induce angiogenesis and defined a new prognostic model. Four CAF angiogenesis-related genes were used for gene- and protein-level validation. Subsequently, CAPS was validated using six independent datasets. In the validation cohorts, this model was closely related to the independent prognosis of patients with GC and immunotherapy efficacy and can be used as a prediction tool for clinical treatment selection and the outcomes of patients with GC.

## Data availability statement

The original contributions presented in the study are included in the article/**Supplementary Material**. Further inquiries can be directed to the corresponding authors.

## Author contributions

YX: Data curation, Formal analysis, Writing – original draft, Writing – review & editing, Software. XW: Data curation, Methodology, Formal analysis, Validation, Visualization, Software, Writing – original draft, Writing – review & editing. JL: Data curation, Software, Writing – original draft, Writing – review & editing. YL: Software, Validation, Writing – original draft, Writing – review & editing. LD: Conceptualization, Validation, Writing – original draft, Writing – review & editing. HW: Writing – original draft, Writing – review & editing, Data curation. XL: Data curation, Formal analysis, Investigation, Writing – original draft, Writing – review & editing. XD: Funding acquisition, Project administration, Supervision, Writing – original draft, Writing – review & editing. QW: Funding acquisition, Supervision, Writing – original draft, Writing – review & editing, Project administration.
