# Supplementary material for: Gastric cancer fibroblasts affect the effect of immunotherapy and patient prognosis by inducing micro-vascular production
Source: Front Immunol. 2024 Jul 8;15:1375013. doi: 10.3389/fimmu.2024.1375013 (PMC11260615; doi:10.3389/fimmu.2024.1375013)
Supplement: Supplementary file 1 [file DataSheet_1.docx]

Supplementary Material

# Supplementary Figure S1. Immune microenvironment analysis (ssGSEA algorithm) and differential expression of prognosis related genes.


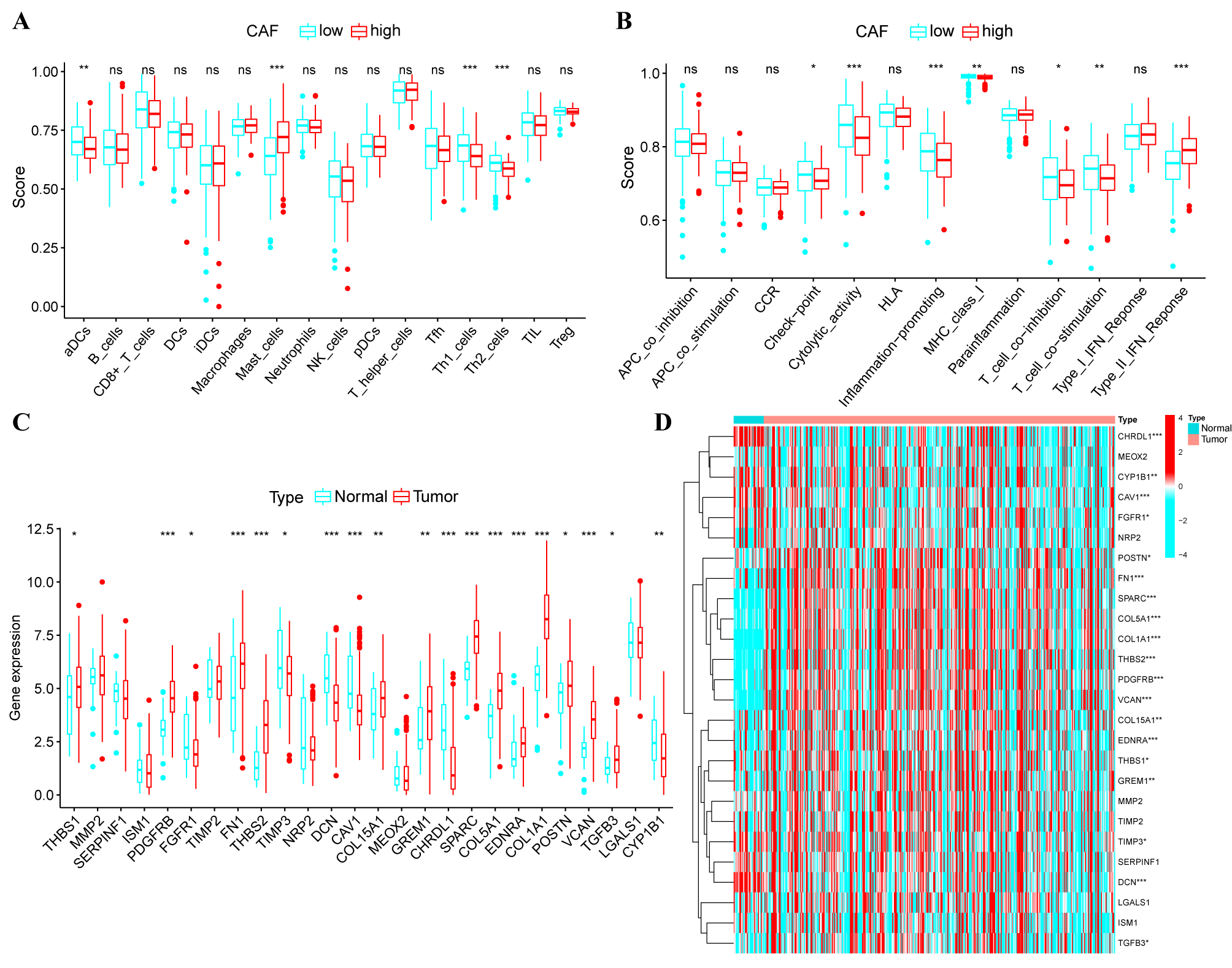


FIGURE S1: Immune microenvironment analysis (ssGSEA algorithm) and differential expression of prognosis related genes. (A) The boxplot of 16 immune cell differences in the low CAF and high CAF groups. (B) The boxplot of 13 immune signaling pathway differences in the low CAF and high CAF groups. (C) The boxplot of differential expression of prognostic related genes. (D) The heatmap of differential expression of prognostic related genes. *p < 0.05, **p < 0.01, ***p < 0.001, ns, p ≥ 0.05.

# Supplementary Figure S2. Correlation analysis between model genes and CAFs.


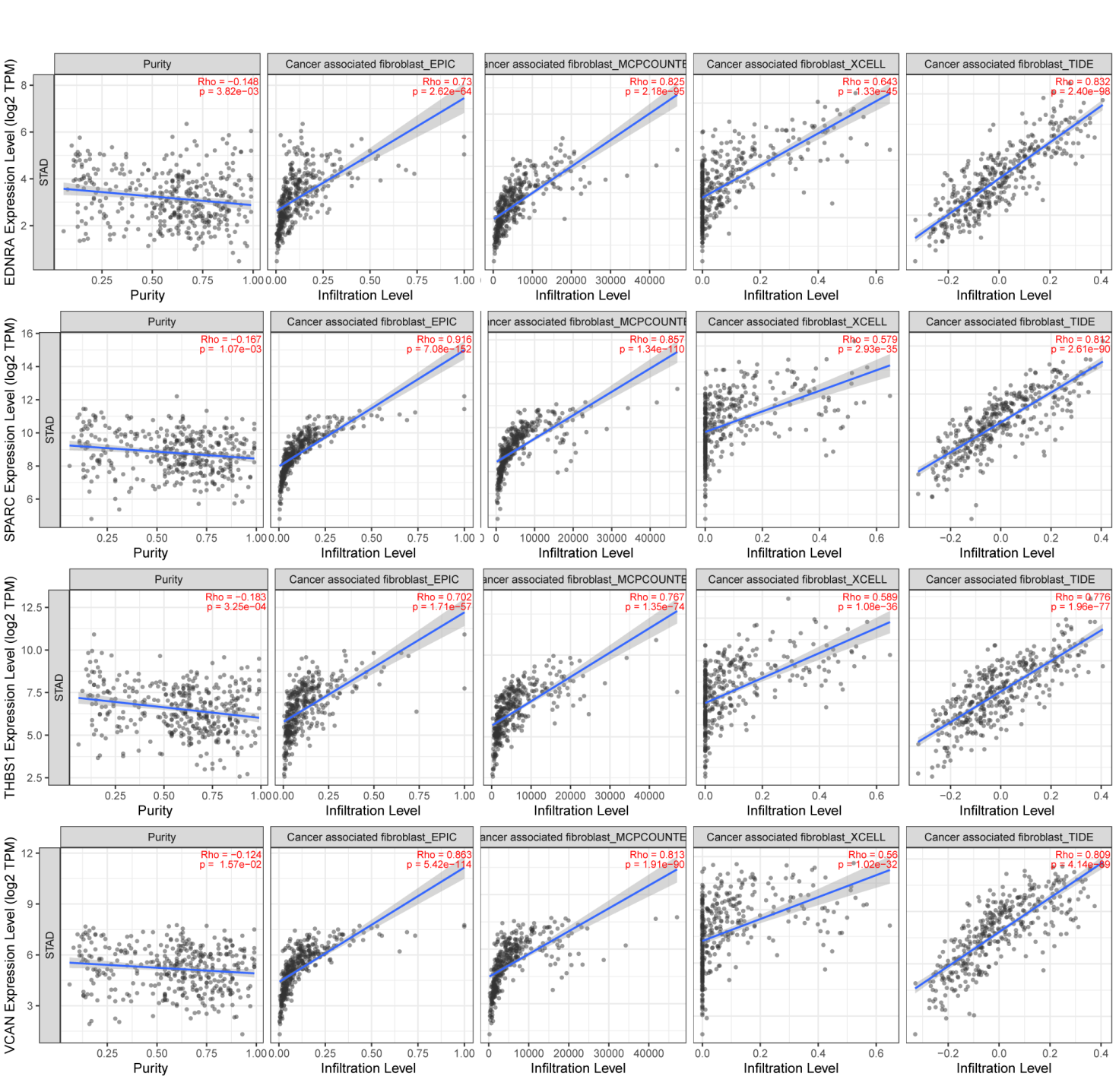


FIGURE S2: Correlation analysis between model genes and CAFs. (A) Based on the TIMER database, four algorithms were used to calculate the correlation between model genes and CAFs.

# Supplementary Figure S3. CAPS was validated in five independent GEO datasets.


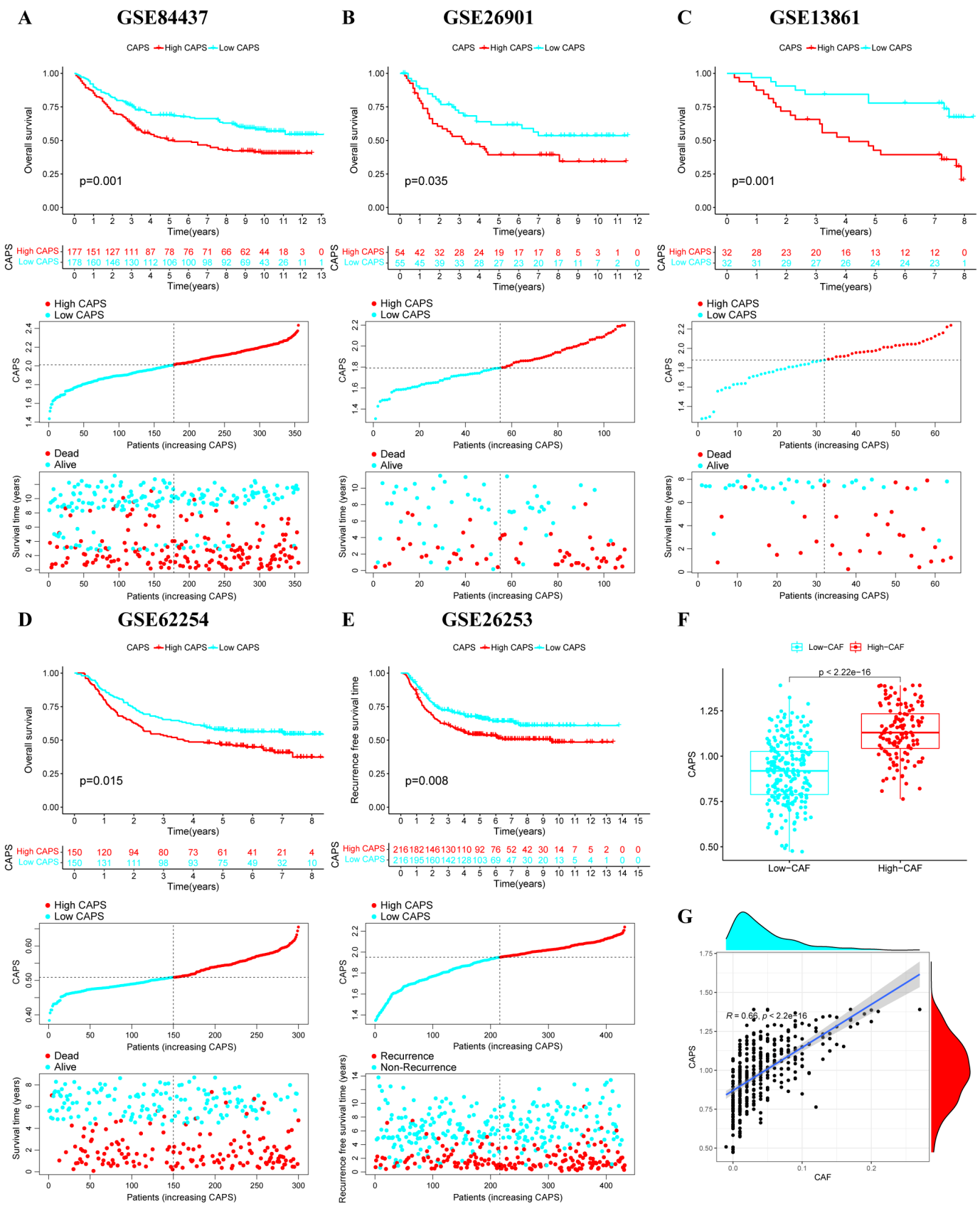


FIGURE S3: CAPS was validated in five independent GEO datasets. In (A) GSE84437, (B) GSE26901, (C) GSE13861, and (D) GSE62254 data sets, Kaplan-Meier survival analysis, distribution of CAPS and survival status analysis were performed in the high and low CAPS groups. (E) Kaplan-meier survival analysis, CAPS distribution, and recurrence status were performed in the GSE26253 dataset. (F) The boxplot of CAPS difference between high and low CAF groups. (G) The scatter plot of correlation between CAPS and CAF score. CAPS, CAF-angiogenesis prognostic score; CAF, cancer-associated fibroblast.

# Supplementary Figure S4. Nomograph Model.


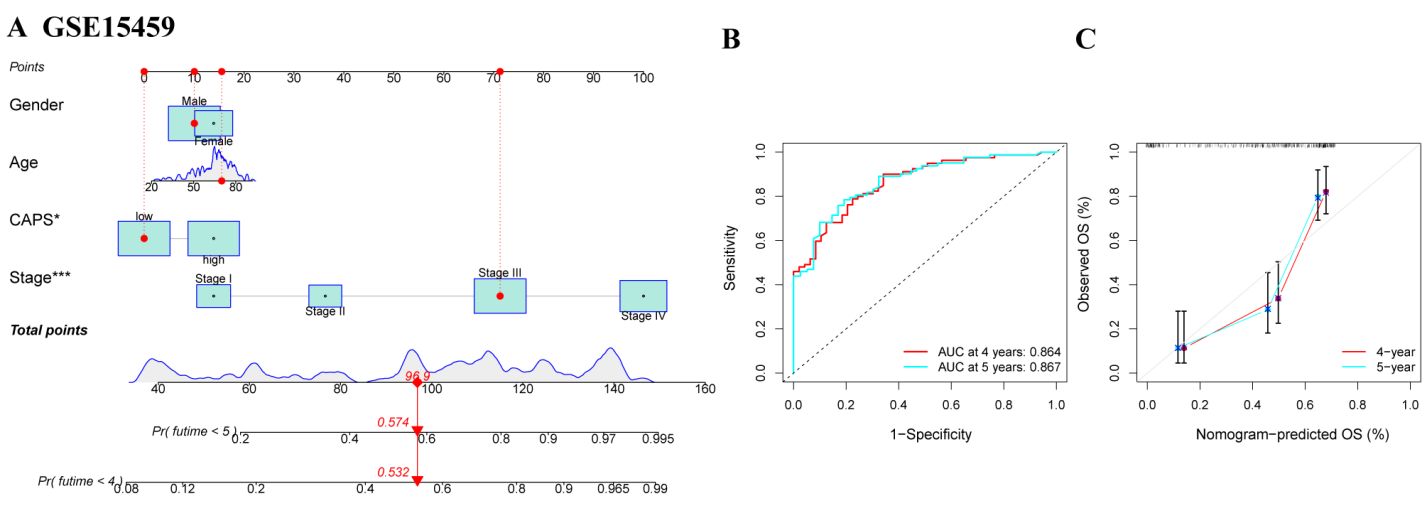


FIGURE S4: Nomograph Model. (A) Nomogram of CAPS and clinical characteristics predicting survival probability of GC patients in GSE15459 cohort. (B) The ROC curve verifies the predictive ability of the nomogram in GSE15459 cohort. (C) Calibration curve for the predictive ability of nomograms in GSE15459 cohort.

# Supplementary Figure S5. Multiple datasets validate the expression of model genes.

#
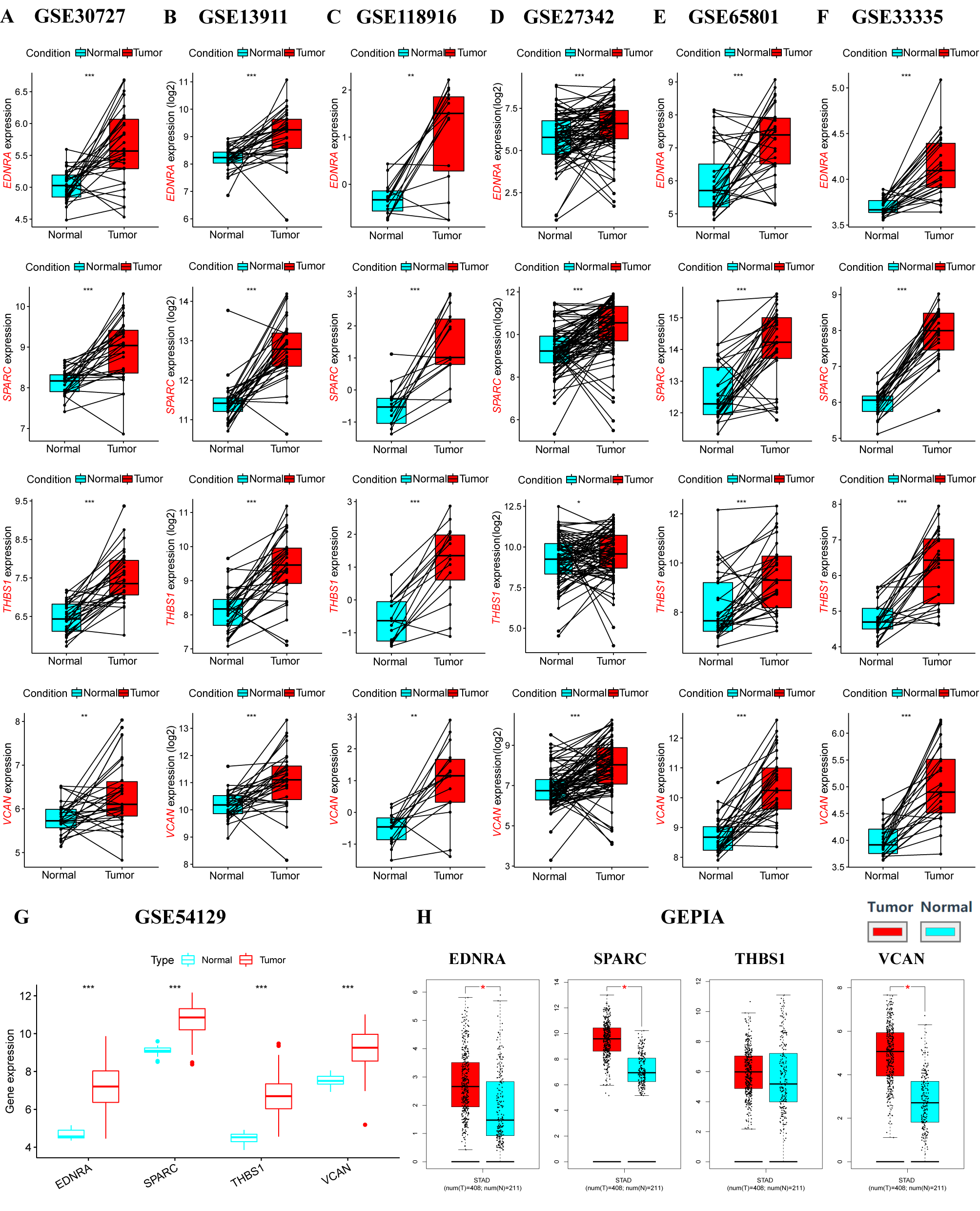


FIGURE S5: Multiple datasets validate the expression of model genes. The expression of model genes in (A) GSE30727, (B) GSE13911, (C) GSE118916, (D) GSE27342, (E) GSE65801, (F) GSE33335, (G) GSE54129 and (H) GEPIA datasets. *p < 0.05, **p < 0.01, ***p < 0.001.

# Supplementary Figure S6. Immune microenvironment (ssGSEA algorithm) and subtype analysis .


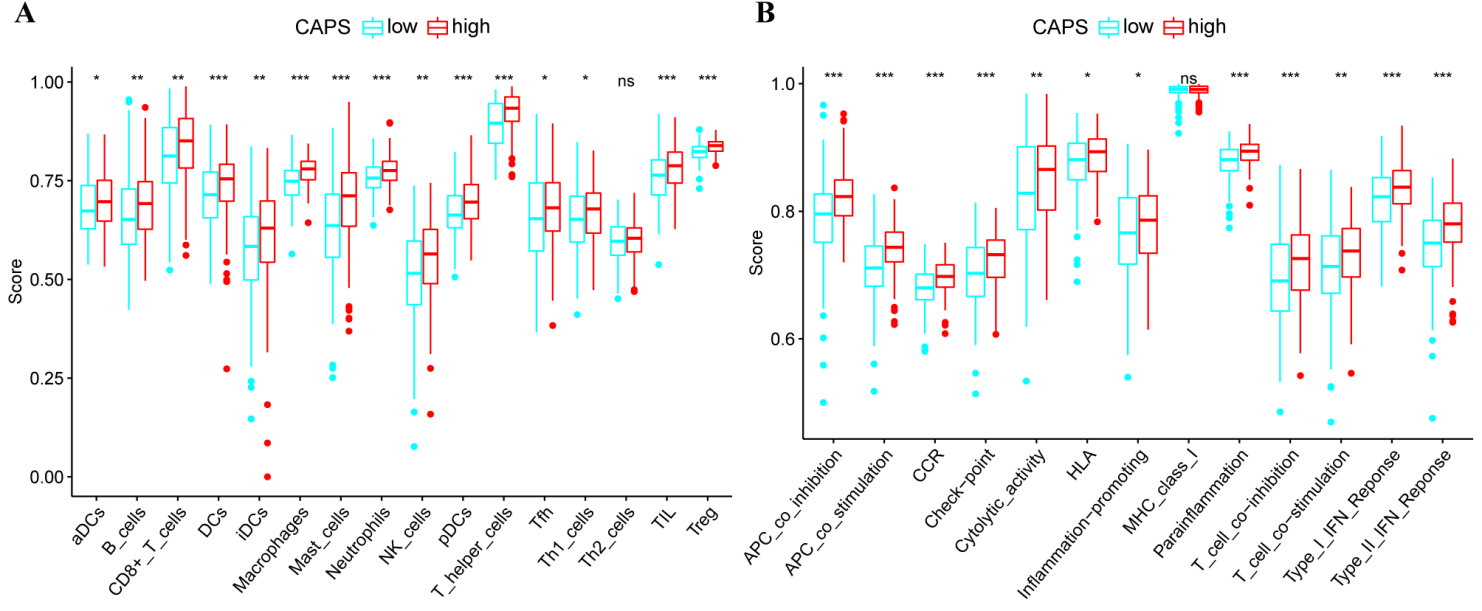


FIGURE S6: Immune microenvironment analysis (ssGSEA algorithm). (A) The boxplot of 16 immune cell differences in the low and high CAPS groups. (B) The boxplot of 13 immune signaling pathway differences in the low and high CAPS groups. ssGSEA, single sample gene set enrichment analysis; CAPS, CAF-angiogenesis prognostic score. *p < 0.05, **p < 0.01, ***p < 0.001, ns, p ≥ 0.05.

# Supplementary Table S1. Patients’ clinical features of TCGA and GEO cohorts.

| Variables | | TCGA (n = 350) | | GSE15459 (n = 191) | | GSE84437 (n = 431) | | GSE26253 (n = 432 ) | |
| --- | --- | --- | --- | --- | --- | --- | --- | --- | --- |
|  |  | Number | Percentage (%) | Number | Percentage (%) | Number | Percentage (%) | Number | Percentage (%) |
| Age | ≥ 60 | 238 | 68.00 | 132 | 69.11 | 249 | 57.77 | 0 | 0.00 |
|  | < 60 | 109 | 31.14 | 59 | 30.89 | 182 | 42.23 | 0 | 0.00 |
|  | Unknow | 3 | 0.86 | 0 | 0.00 | 0 | 0.00 | 0 | 0.00 |
| Gender | Male | 226 | 64.57 | 124 | 64.92 | 294 | 68.21 | 0 | 0.00 |
|  | Female | 124 | 35.43 | 67 | 35.08 | 137 | 31.79 | 0 | 0.00 |
| Survival status | Alive | 207 | 59.14 | 96 | 50.26 | 224 | 51.97 | 255 | 59.16 |
|  | Dead | 143 | 40.86 | 95 | 49.74 | 207 | 48.03 | 177 | 41.07 |
| Grade | G1 | 9 | 2.57 | 0 | 0.00 | 0 | 0.00 | 0 | 0.00 |
|  | G2 | 125 | 35.71 | 0 | 0.00 | 0 | 0.00 | 0 | 0.00 |
|  | G3 | 207 | 59.14 | 0 | 0.00 | 0 | 0.00 | 0 | 0.00 |
|  | Unknow | 9 | 2.57 | 0 | 0.00 | 0 | 0.00 | 0 | 0.00 |
| Clinical stage | Stage I | 46 | 13.14 | 31 | 16.23 | 0 | 0.00 | 68 | 15.74 |
|  | Stage II | 110 | 31.43 | 29 | 15.18 | 0 | 0.00 | 167 | 38.66 |
|  | Stage III | 145 | 41.43 | 72 | 37.70 | 0 | 0.00 | 130 | 30.09 |
|  | Stage IV | 35 | 10.00 | 59 | 30.89 | 0 | 0.00 | 67 | 15.51 |
|  | Unknow | 14 | 4.00 | 0 | 0.00 | 0 | 0.00 | 0 | 0.00 |
| Tstage | T1 | 16 | 4.57 | 0 | 0.00 | 11 | 2.55 | 0 | 0.00 |
|  | T2 | 74 | 21.14 | 0 | 0.00 | 38 | 8.82 | 0 | 0.00 |
|  | T3 | 161 | 46.00 | 0 | 0.00 | 92 | 21.35 | 0 | 0.00 |
|  | T4 | 95 | 27.14 | 0 | 0.00 | 290 | 67.29 | 0 | 0.00 |
|  | Unknow | 4 | 1.14 | 0 | 0.00 | 0 | 0.00 | 0 | 0.00 |
| Mstage | M0 | 312 | 89.14 | 0 | 0.00 | 0 | 0.00 | 0 | 0.00 |
|  | M1 | 23 | 6.57 | 0 | 0.00 | 0 | 0.00 | 0 | 0.00 |
|  | Unknow | 15 | 4.29 | 0 | 0.00 | 0 | 0.00 | 0 | 0.00 |
| Nstage | N0 | 103 | 29.43 | 0 | 0.00 | 80 | 18.56 | 0 | 0.00 |
|  | N1 | 93 | 26.57 | 0 | 0.00 | 187 | 43.39 | 0 | 0.00 |
|  | N2 | 72 | 20.57 | 0 | 0.00 | 132 | 30.63 | 0 | 0.00 |
|  | N3 | 71 | 20.29 | 0 | 0.00 | 32 | 7.42 | 0 | 0.00 |
|  | unknow | 11 | 3.14 | 0 | 0.00 | 0 | 0.00 | 0 | 0.00 |
|  |  |  |  |  |  |  |  |  |  |
| Variables | | GSE13861 (n = 64) | | GSE66254 (n = 300) | | GSE26901 (n = 109) | |  |  |
|  |  | Number | Percentage (%) | Number | Percentage (%) | Number | Percentage (%) |  |  |
| Age | ≥ 60 | 40 | 62.50 | 194 | 64.67 | 45 | 41.28 |  |  |
|  | < 60 | 24 | 37.50 | 106 | 35.33 | 64 | 58.72 |  |  |
|  | Unknow | 0 | 0.00 | 0 | 0.00 | 0 | 0.00 |  |  |
| Gender | Male | 45 | 70.31 | 199 | 66.33 | 69 | 63.30 |  |  |
|  | Female | 19 | 29.69 | 101 | 33.67 | 40 | 36.70 |  |  |
| Survival status | Alive | 33 | 51.56 | 148 | 49.33 | 54 | 49.54 |  |  |
|  | Dead | 31 | 48.44 | 152 | 50.67 | 55 | 50.46 |  |  |
| Grade | G1 | 0 | 0.00 | 0 | 0.00 | 0 | 0.00 |  |  |
|  | G2 | 0 | 0.00 | 0 | 0.00 | 0 | 0.00 |  |  |
|  | G3 | 0 | 0.00 | 0 | 0.00 | 0 | 0.00 |  |  |
|  | Unknow | 0 | 0.00 | 0 | 0.00 | 0 | 0.00 |  |  |
| Clinical stage | Stage I | 12 | 18.75 | 30 | 10.00 | 40 | 36.70 |  |  |
|  | Stage II | 11 | 17.19 | 97 | 32.33 | 18 | 16.51 |  |  |
|  | Stage III | 25 | 39.06 | 96 | 32.00 | 36 | 33.03 |  |  |
|  | Stage IV | 16 | 25.00 | 77 | 25.67 | 15 | 13.76 |  |  |
|  | Unknow | 0 | 0.00 | 0 | 0.00 | 0 | 0.00 |  |  |
| Tstage | T1 | 0 | 0.00 | 0 | 0.00 | 0 | 0.00 |  |  |
|  | T2 | 0 | 0.00 | 188 | 62.67 | 0 | 0.00 |  |  |
|  | T3 | 0 | 0.00 | 91 | 30.33 | 0 | 0.00 |  |  |
|  | T4 | 0 | 0.00 | 21 | 7.00 | 0 | 0.00 |  |  |
|  | Unknow | 0 | 0.00 | 0 | 0.00 | 0 | 0.00 |  |  |
| Mstage | M0 | 55 | 85.94 | 273 | 91.00 | 102 | 93.58 |  |  |
|  | M1 | 4 | 6.25 | 27 | 9.00 | 7 | 6.42 |  |  |
|  | Unknow | 5 | 7.81 | 0 | 0.00 | 0 | 0.00 |  |  |
| Nstage | N0 | 0 | 0.00 | 38 | 12.67 | 0 | 0.00 |  |  |
|  | N1 | 0 | 0.00 | 131 | 43.67 | 0 | 0.00 |  |  |
|  | N2 | 0 | 0.00 | 80 | 26.67 | 0 | 0.00 |  |  |
|  | N3 | 0 | 0.00 | 51 | 17.00 | 0 | 0.00 |  |  |
|  | unknow | 0 | 0.00 | 0 | 0.00 | 0 | 0.00 |  |  |
